# The role of ubiquitin ligases in regulating immune cell functions

**DOI:** 10.3389/fimmu.2025.1625419

**Published:** 2025-10-15

**Authors:** Saci-Elodie Marty, Linda Yip, Fangyuan Wang, Manoj Kumar, C. Garrison Fathman

**Affiliations:** Department of Medicine, School of Medicine, Stanford University, Palo Alto, CA, United States

**Keywords:** neddylation, regulatory T cell (Treg), cullin ring ligases (CRLs), IL-2 receptorsignaling, pJAK1 degradation, GRAIL, neddylation activating enzyme inhibitor (NAEi)

## Abstract

Regulatory T cells (Tregs) play a central role in immune homeostasis and the preservation of immunological self-tolerance. Treg activity depends on prolonged IL-2 receptor (IL-2R) signaling, and impairment or loss of this function has been linked to the development of autoimmune diseases. This review evaluates the hypothesis that disrupted IL-2R signaling, due to enhanced desensitization, impairs Treg suppressive function and contributes to autoimmunity. In mice and humans, desensitization of IL-2R signaling by the cullin-RING ligase 5 (CRL5) complex leads to reduced persistence of phosphorylated JAK1 (pJAK1) and its downstream effector pSTAT5, a transcription factor critical for Treg function. Activation of CRL5 requires neddylation—a post-translational modification in which the ubiquitin-like NEDD8 is conjugated to lysine 724 on cullin-5 (CUL5), the scaffold protein of CRL5. Neddylation permits untethering of the RING-box protein RBX, enabling E2 enzyme-mediated ubiquitination and proteasomal degradation of pJAK1 via recruitment by suppressor of cytokine signaling 3 (SOCS3). This process, known as IL-2R signal desensitization, is antagonized in Tregs by the E3 ligase GRAIL (Gene Related to Anergy in Lymphocytes, RNF128), which mono-ubiquitinates Lys724 to block neddylation, preventing CRL5 activation and pJAK1 degradation. An imbalance between neddylation and mono-ubiquitination at Lys724 compromises IL-2R signaling and promotes autoimmune pathology, and studies show GRAIL expression is diminished in Tregs from autoimmune patients and mouse models, leading to reduced pSTAT5 activity and impaired suppressive capacity. Pharmacologic inhibition of neddylation with pathway inhibitors (NAEi) restores IL-2R signaling and Treg function, highlighting the therapeutic potential of targeting this regulatory axis to preserve immune tolerance.

## 1 Introduction

Regulatory T cells (Tregs) are a subset of CD4^+^ T cells that recognize self-antigens and function to suppress immune responses directed against autologous tissues, particularly at sites of inflammation or tissue injury, thereby preventing autoimmunity. Defective Treg function has been implicated as a contributing factor in a variety of autoimmune diseases, including systemic lupus erythematosus (SLE) (1), type 1 diabetes (T1D) (2, 3), and rheumatoid arthritis (RA) (4). Similar Treg dysfunction has also been observed in patients with celiac disease and Graves’ disease (5, 6). Although reduced frequencies of Tregs have been reported in some autoimmune conditions, efforts to expand autologous Treg populations have often failed to improve clinical outcomes, likely due to persistent functional impairments. Therefore, therapeutic strategies aimed at restoring Treg function rather than increasing Treg numbers may be more effective (7, 8).

For example, low-dose IL-2 therapy in patients with Sjögren’s syndrome led to a four-fold increase in peripheral Tregs but did not result in measurable improvement in disease activity (9). In T1D and multiple sclerosis (MS), Treg frequencies are not significantly different from healthy controls, but these cells do exhibit a reduced capacity to suppress effector T cell proliferation (2, 3, 10). Collectively, these findings support the notion that restoring endogenous Treg function, rather than increasing their abundance, represents a more effective approach for preventing autoimmunity.

Proper Treg function is critically dependent on interleukin-2 receptor (IL-2R) signaling and mTOR (mechanistic target of rapamycin) pathway regulation (11, 12). Defective IL-2R signaling is a shared immunological phenotype across multiple autoimmune diseases. Genome-wide association studies have identified multiple risk alleles in the IL-2/IL-2R signaling pathway that are associated with increased susceptibility to diseases such as T1D, MS, SLE, RA, Crohn’s disease, Graves’ disease, and alopecia areata (13).

Evidence from both human and murine studies has shown diminished IL-2R signaling, decreased pSTAT5 phosphorylation, and reduced FOXP3 expression in Tregs from autoimmune individuals (13–15), suggesting multiple mechanisms may contribute to this signaling defect. Sustained IL-2R signaling, maintained for at least four hours, is necessary to support Treg suppressive function (16, 17). One mechanism contributing to early termination of IL-2R signaling involves decreased expression of the Gene Related to Anergy in Lymphocytes (GRAIL), an E3 ubiquitin ligase essential for Treg function (18). Forced expression of GRAIL can induce a regulatory phenotype in conventional T cells (19), and GRAIL-deficient mice exhibit impaired tolerance induction and increased susceptibility to autoimmune disease (20).

In non-obese diabetic (NOD) mice, Tregs exhibit IL-2R signaling defects (21), which can be partially restored with IL-2 treatment (22). However, IL-2 elicits a significantly lower pSTAT5 response in the Tregs from these mice compared to control mice (23). Additionally, the Tregs of NOD mice express significantly lower amounts of GRAIL mRNA and protein compared to controls (23), suggesting that reduced GRAIL expression and defective IL-2R signaling contribute to the development of disease in NOD mice.

Evidence indicates that reduced GRAIL expression disrupts the inhibition of IL-2R desensitization, thereby impairing Treg function. This supports the rationale that therapeutic restoration of Treg activity, rather than numerical enhancement alone, is necessary for effective treatment of autoimmune diseases. While interventions such as low-dose IL-2, Treg adoptive transfer, or combined therapies can transiently increase Treg frequency, they often fail to achieve durable clinical efficacy. For instance, IL-2/rapamycin therapy in T1D patients augmented circulating Tregs but unexpectedly led to impaired β-cell function (24). In another study, low-dose IL-2 increased both Tregs and CD8^+^/NK cells which are implicated in β-cell destruction in T1D (25–27).

Based on these observations, therapeutic efforts should prioritize the recovery of endogenous Treg function. The following sections examine how post-translational modifications, particularly ubiquitination and neddylation, regulate Treg function via GRAIL and downstream signaling intermediates. In particular, the interplay between GRAIL and CRLs, and its effect on the JAK-STAT and mTOR signaling pathways, is explored as a mechanistic basis for inhibiting IL-2R desensitization and enhancing/restoring Treg function. This review also discusses how targeting this axis using small-molecule inhibitors of the neddylation pathway may offer a strategy to correct dysfunctional IL-2R signaling and restore endogenous Treg function in autoimmune diseases.

## 2 Ubiquitination and neddylation

### 2.1 Ubiquitination: enzyme cascade

Ubiquitination is a post-translational modification that plays a key role in protein turnover and immune regulation, including the maintenance of Treg suppressive function. It involves the covalent attachment of a small 76 amino acid protein Ubiquitin (Ub) to a target substrate. The type of ubiquitination determines the fate of the modified protein. Polyubiquitination, attachment of a ubiquitin chain to a single lysine residue, targets proteins for proteasomal degradation (28), while mono-ubiquitination, attachment of a single ubiquitin to a lysine residue, regulates protein localization and function (29).

Ubiquitin (Ub) is transferred to a target protein through a highly conserved three-step enzyme cascade: E1 Ubiquitin-activating enzyme, E2 Ubiquitin-conjugating enzyme, and E3 Ubiquitin ligase enzyme. Energy from ATP is used to fuel the formation of a thioester bond between E1 and Ub. Ub is then passed to E2 forming a second thioester bond. E3 ligases facilitate the transfer of Ub from E2 to the lysine of a target protein forming a stable isopeptide bond between Ub and the target substrate (30). There are three main E3 ligases: RING-type, HECT-type, and RBR-type. RING-type ligases mediate a direct transfer of Ub from E2 to the substrate. HECT-type ligases have an intermediary step before transferring Ub to the substrate. RBR-type ligases are a combination of the previous mechanisms mentioned (28).

Within Tregs, multiple ubiquitination events occur across critical signaling pathways. For instance, polyubiquitination at K48 and K63 sites targets intermediates in the JAK–STAT and mTOR pathways for degradation (30). In contrast, mono-ubiquitination contributes to IL-2 receptor sensitivity, intracellular trafficking, and transcriptional stability, all of which are necessary to maintain Treg suppressive function. At specific sites such as Lys724, mono-ubiquitination can also sterically block neddylation, since occupation by Ub prevents additional modification and creates local structural interference (29).

Many E3 ligases, particularly RING-type, can undergo autoubiquitination, which serves as a self-regulatory mechanism to modulate their own stability and activity (31). Some ligases evade autoubiquitination through structural constraints such as a lack of lysine residues in substrate-binding domains, which limits Ub attachment.

Ubiquitination is counteracted by deubiquitinating enzymes (DUBs), which remove Ub moieties and help preserve protein stability and signaling fidelity (32). In Tregs, the DUB USP21 deubiquitinates and stabilizes GATA3 and FOXP3, both of which are central to Treg differentiation and suppressive activity (33, 34). By maintaining FOXP3 expression, USP21 prevents lineage destabilization and limits conversion to Th1-like effector cells (34). Other DUBs, including USP8, Otub1, and Otub1-ARF1, also contribute to Treg regulation and will be discussed in later sections.

While DUBs provide a dynamic counterbalance to ubiquitin ligases, neddylation, another post-translational pathway, specifically regulates the activity of cullin-RING ligases (CRLs), which are crucial for controlling IL-2R signaling and Treg function.

### 2.2 Neddylation: activating cullin-RING ligases

Neddylation is a ubiquitin-like post-translational modification that regulates the activity of cullin-RING ligases (CRLs), a family of E3 ubiquitin ligases involved in immune signaling and protein turnover. This pathway centers on Neural precursor cell Expressed Developmentally Down-regulated protein 8 (NEDD8). NEDD8 is an 81–amino acid ubiquitin-like protein that becomes active once C-terminal hydrolase exposes Gly76 (35).

Like ubiquitin, NEDD8 is transferred through a three-enzyme cascade involving E1, E2, and E3 enzymes. The neddylation cascade starts with NEDD8-Activating Enzyme E1 (NAE), a heterodimer made up of UBA3/NAEβ (catalytic subunit) and APPBP1/NAE1 (regulatory subunit) (36). NAE recognizes NEDD8 and, fueled by the conversion of ATP to AMP, activates its C-terminal diglycine. NEDD8 binds to the adenylation site on UBA3 and forms a temporary thioester bond at the cysteine transthiolation domain. The linking of NEDD8 to NAE converts the enzyme from an open conformation to a closed state as NEDD8 binding to cysteine brings the domains closer. The C-terminal ubiquitin fold domain (UFD) on UBA3 also plays a role in recruiting the next enzyme in the cascade. The NEDD8 Conjugating Enzyme E2, either UBE2M or UBE2F, binds to NAE through its interaction with UFD on UBA3. Once NAE is double-loaded with NEDD8, a structural change caused by neddylation allows E2 to temporarily interact with E1. This interaction brings E2’s cysteine close to E1’s active site, enabling NEDD8 to be transferred from E1 to E2. After the transfer, E2-NEDD8 detaches from E1 and begins the third step in the cascade. The E2–NEDD8 complex then interacts with the cullin scaffold of CRLs to transfer NEDD8 onto a conserved lysine residue of the target substrate completing the process.

Neddylation induces a structural rearrangement in CRLs, enhancing their ubiquitin ligase activity. This modification promotes untethering of the RING-box protein (RBX) from the cullin scaffold, bringing the E2 ubiquitin-conjugating enzyme into proximity with substrate-bound SOCS adaptors and enabling efficient ubiquitin transfer. Additionally, neddylation prevents binding of CAND1, a negative regulator that inhibits inactive CRLs (36).

The primary targets of neddylation are cullin family proteins, which include CUL1 through CUL9, each with distinct tissue distributions and pathway specificities. In Tregs, the relevant neddylation cascade involves the UBE2F–RBX2 complex and CUL5, which plays a central role in IL-2 receptor (IL-2R) desensitization through degradation of pJAK1.

Although over 300 CRL complexes have been described based on various combinations of cullin and RING proteins (37), not all are relevant in the immune system or in Treg regulation. Neddylation in this context specifically modulates CRL5 activity, which controls cytokine receptor signaling thresholds and CRL1 that activates mTOR, both contribute to the maintenance or breakdown of immune tolerance.

## 3 Post-translational regulation of Treg function

Treg function and immune system balance are tightly controlled by post-translational modifications that fine-tune key signaling pathways. In Tregs, ubiquitination and neddylation serve as crucial modulators of IL-2 signaling and mTOR pathway regulation.

Ubiquitination by E3 ligases marks proteins for proteasomal degradation and can also regulate signaling intermediates through mono-ubiquitination, which affects protein localization, stability, and function (18). This form of regulation is particularly important in Tregs, where precise control of IL-2 receptor (IL-2R) signaling is essential to maintain immune tolerance and prevent the development of autoimmunity.

Cullin-RING ligases (CRLs), a large family of E3 ubiquitin ligases, are involved in multiple immune regulatory processes. A key member of this family, CRL5, is activated by neddylation, a post-translational modification involving conjugation of the ubiquitin-like protein NEDD8 to a conserved lysine residue (Lys724) on the scaffold protein cullin-5 (CUL5). This modification induces a conformational change that enables E2 enzyme–mediated ubiquitination and subsequent degradation of IL-2R signaling intermediates such as pJAK1 (36).

Prolonged IL-2R signaling is required to stabilize the Treg phenotype and support FOXP3-dependent suppressive function. This process is negatively regulated by CRL5-mediated degradation of pJAK1. To counteract this, GRAIL functions as a regulatory E3 ligase that blocks IL-2R desensitization. GRAIL mono-ubiquitinates Lys724 on CUL5, directly competing with NEDD8 and thereby preventing CRL5 activation. By doing so, GRAIL preserves IL-2R signaling, prolongs pSTAT5 phosphorylation, and sustains the transcription of genes necessary for Treg suppressive activity.

Due to structural similarities between CUL1 and CUL5, it is likely that GRAIL also modulates mTOR signaling by ubiquitinating Lys720 on CUL1, thereby protecting DEPTOR, an endogenous mTOR inhibitor, from CRL1-mediated degradation. This dual mechanism prevents hyperactivation of mTORC1 to maintain the quiescent metabolic profile characteristic of functional Tregs. It is hypothesized that in the absence of GRAIL, both pJAK1 and DEPTOR are degraded, disrupting IL-2R signaling that contributes to Treg dysfunction and subsequent autoimmunity.

When GRAIL expression is diminished, neddylation proceeds unchecked, leading to CRL5-mediated degradation of IL-2R second messengers and CRL1 degradation of DEPTOR. This results in shortened IL-2R signaling, decreased pSTAT5 activation, increased activity of mTORC1, and ultimately, diminished Treg suppressive function. These changes contribute to immune dysregulation and increased susceptibility to autoimmunity.

## 4 IL-2 receptor signaling pathway

The interleukin-2 receptor (IL-2R) is a heterotrimeric cell surface receptor composed of three subunits: IL-2Rα (CD25), IL-2Rβ (CD122), and IL-2Rγ (CD132) (38). IL-2 is critical for the survival, proliferation, and suppressive function of Tregs. Although Tregs are incapable of producing IL-2 due to FOXP3-mediated transcriptional repression of the IL-2 gene (39), they express CD25 (α subunit), which forms the high-affinity IL-2R complex. This allows Tregs to efficiently respond to IL-2 produced by neighboring activated T cells in the local microenvironment.

Due to their constitutive expression of the high-affinity IL-2R, Tregs can respond to subnanomolar concentrations of IL-2. However, under subnanomolar concentrations effector T cells (Teffs) which transiently express IL-2Rα upon activation, remain unresponsive as they do not permanently express IL-2Rα.

**Figure 1:**
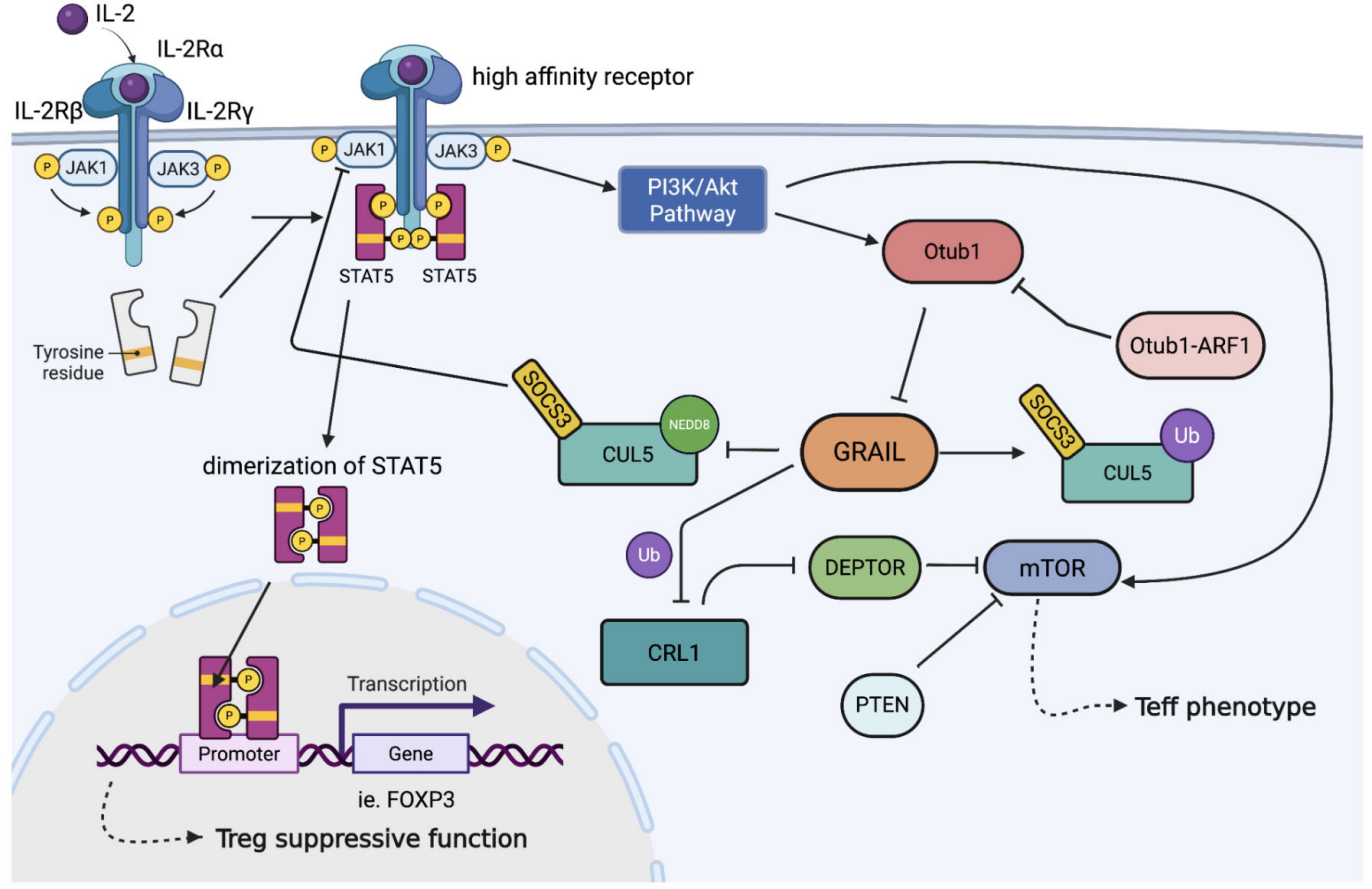
Regulation of IL-2R signaling and Treg stability by post-translational modification of CRL5. Upon IL-2 binding to its high-affinity receptor (IL-2Rα/β/γ), JAK1 is activated and phosphorylates STAT5, leading to its dimerization and nuclear translocation to drive FOXP3 transcription and Treg suppressive function. SOCS3 recruits the CRL5 complex to ubiquitinate phosphorylated JAK1 (pJAK1). Neddylation activates SOCS3-CRL5 interaction, allowing the termination of IL-2 signaling. GRAIL counteracts this by mono-ubiquitinating CUL5, preventing neddylation, and thereby sustaining IL-2 signaling. GRAIL also protects DEPTOR from CRL1-mediated degradation, suppressing mTOR activation. Loss of GRAIL or upregulation of the Otub1/ARF1 axis promotes mTOR activity and destabilizes the Treg phenotype. The competitive interplay between neddylation and GRAIL-mediated mono-ubiquitination controls cytokine signaling and regulates Tregs phenotype.

Upon IL-2 binding, the receptor complex undergoes a conformational change that facilitates activation of the Janus kinases JAK1 and JAK3, leading to the phosphorylation of signal transducer and activator of transcription 5 (STAT5) (40) (**Figure 1**). Activated STAT5 (pSTAT5) dimerizes and translocates to the nucleus to drive transcription of key genes involved in Treg identity and function, including FOXP3, IL2RA, and CTLA4 (41). Importantly, IL-2R signaling must be sustained for several hours to maintain adequate transcriptional output and ensure Treg suppressive function (16, 17).

In addition to activating the JAK–STAT pathway, IL-2 also stimulates the PI3K–Akt–mTOR signaling axis, which has different effects depending on T cell subtype (42). In Teffs, IL-2-induced mTORC1 activation enhances glycolytic metabolism and proliferation, thereby promoting effector responses (43). In contrast, excessive mTORC1 activation in Tregs destabilizes their phenotype and impairs suppressive capacity, promoting a shift toward a Teff-like state (43, 44). Therefore, tight regulation of mTOR signaling is critical for maintenance of Treg function.

**Figure 2:**
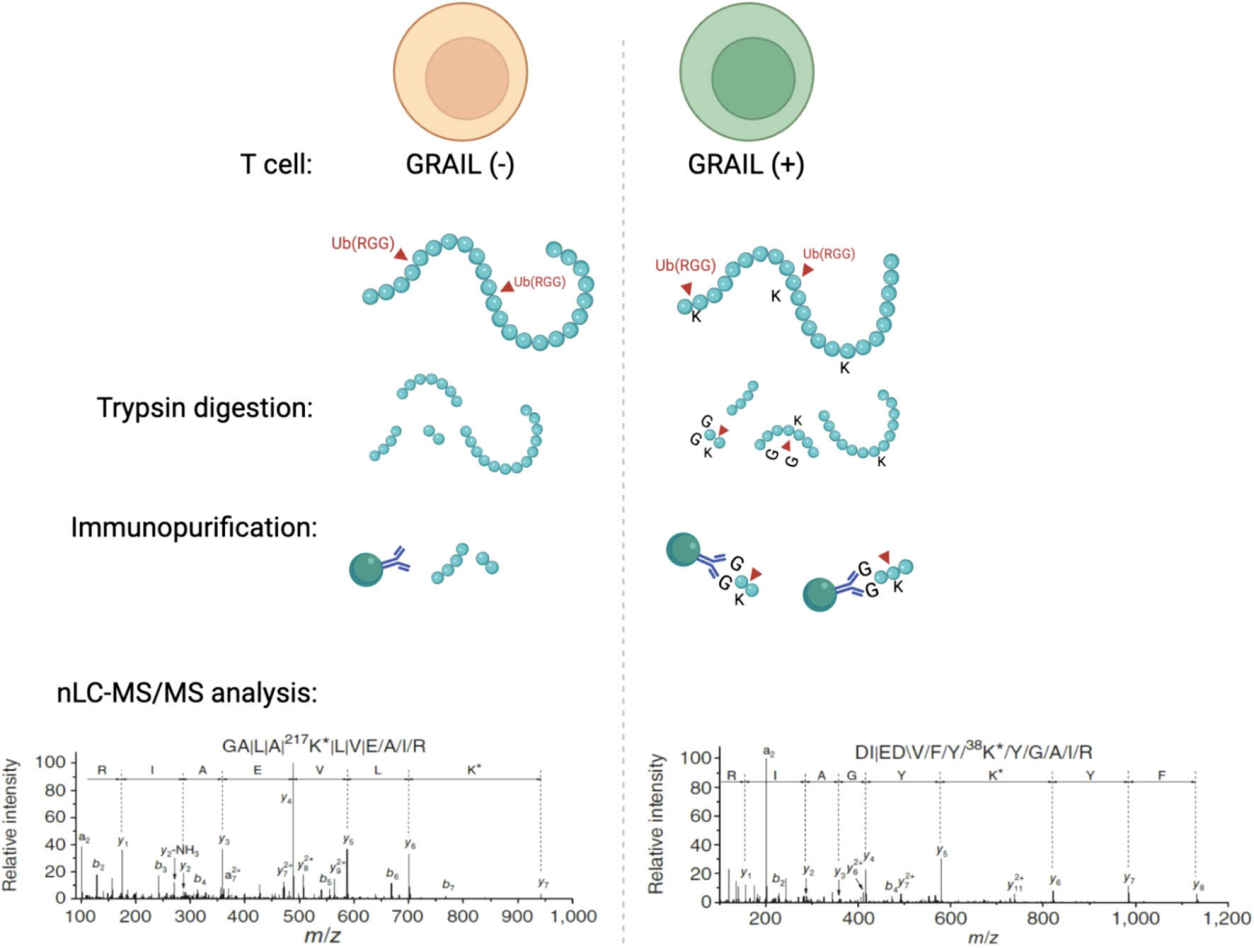
Mass spectrometry and sequencing data shows that GRAIL mono-ubiquitinates CUL5 at Lys724 to inhibit neddylation and suppress CRL5 activity in T cells. Using an E3 target identification system with a monoclonal antibody that recognizes di-glycine tags on ubiquitinated lysines, this study showed that Lys724 on CUL5 was a direct mono-ubiquitination target of GRAIL. This site is normally required for NEDD8 conjugation. By mono-ubiquitinating this exact lysine, GRAIL prevents neddylation and locks the CRL5 complex in an inactive conformation.

This regulatory balance is achieved in part through the actions of GRAIL, which modulates both IL-2R and mTOR signaling. GRAIL antagonizes the CRL5 complex by mono-ubiquitinating Lys724 on CUL5, preventing its neddylation and subsequent activation (**Figure 2**). This inhibits degradation of pJAK1 and enables prolonged STAT5 activation, thereby supporting the transcriptional program required for Treg suppressive function (45).

## 5 CRL5-mediated cytokine desensitization via SOCS3 in Tregs

Suppressors of cytokine signaling (SOCS) proteins function as feedback inhibitors of inflammatory cytokine signaling making them integral to the regulation of immune homeostasis. There are eight SOCS proteins from SOCS1–7 and CIS (46). The basic structure of all SOCS is a C-terminal SOCS box domain that participates in the CRL5 E3 ubiquitin ligase complex and a central Src-homology 2 (SH2) domain which through phosphorylated receptors bind target substrates. Each SOCS is unique at the N-terminal domain. Among the eight SOCS family members, SOCS1 and SOCS3 are unique in containing a kinase inhibitory region (KIR) that enables direct inhibition of JAK kinases (47). In Tregs, SOCS3 acts as a substrate adaptor for the CRL5 E3 ubiquitin ligase complex by binding to phosphorylated JAK1 (pJAK1) through its SH2 domain and tethering it to CRL5 via its SOCS box.

Overexpression of SOCS3 has been shown to accelerate IL-2R desensitization in Tregs, thereby limiting STAT5 activation and impairing suppressive function (23). CRL5-mediated degradation of pJAK1 is activated by neddylation of Lys724 on CUL5, which induces a conformational change that brings the E2 enzyme into close proximity with the substrate–SOCS3 complex (18). This approximately 50 Å structural shift facilitates efficient ubiquitin transfer and promotes pJAK1 degradation (18).

**Figure 3:**
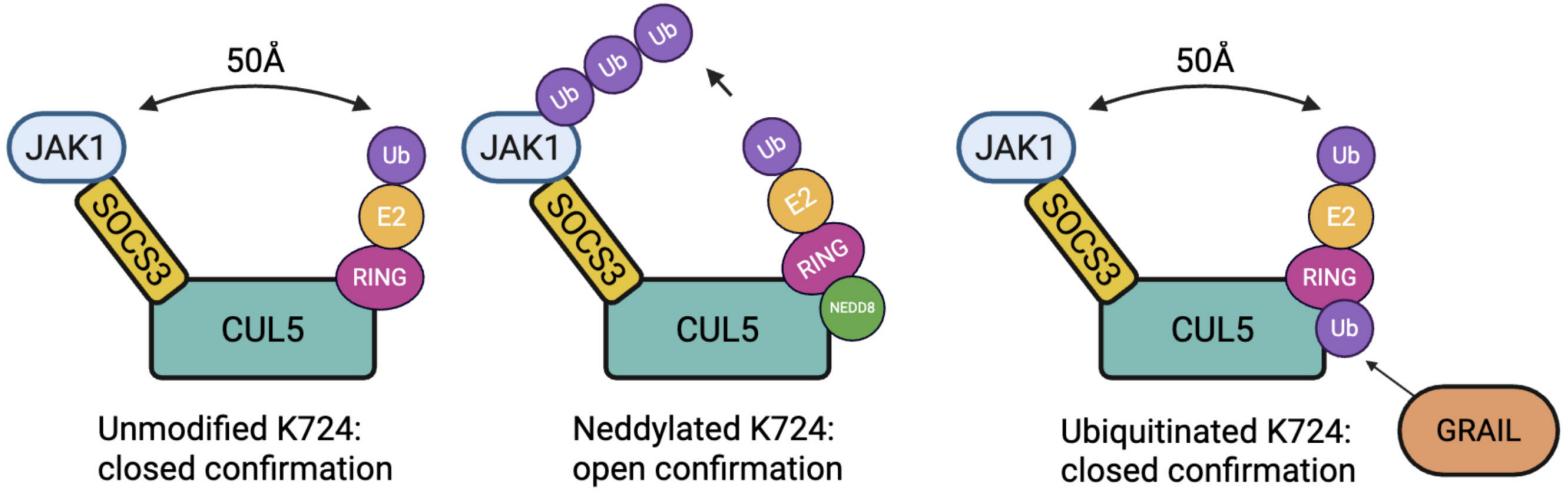
Conformational states of the CRL5 E3 ligase complex modulated by post-translational modification of CUL5 at Lys724. In the unmodified state (left), the complex remains in a closed conformation, preventing effective ubiquitin transfer. Upon neddylation at Lys724 (middle), the CUL5 complex undergoes a ~50 Å shift into an open conformation. Mono-ubiquitination of Lys724 (right), mediated by GRAIL, competes with neddylation and transforms the complex into a closed conformation, inhibiting CRL5 activity and maintaining IL-2R signaling.

In the absence of neddylation, the CRL5 complex remains in an inactive state, as E2 remains spatially separated from the bound substrate. Neddylation thus acts as a molecular hinge, reconfiguring the architecture of the CRL5 complex to permit substrate modification. The modification involves RBX1, a small protein held at the C-terminal end of CUL5 that controls the E2 ubiquitin transferase, to move it into proximity of SOCS3 to allow ubiquitin transfer to the pJAK1 protein bound by SOCS3 (48) (**Figure 3**).

Degradation of pJAK1 prematurely terminates IL-2 signaling, reducing downstream pSTAT5 activity and diminishing FOXP3 expression. Both of which are essential for maintaining Treg activity. Simultaneously, CRL1-mediated degradation of DEPTOR, an inhibitor of mTORC1, leads to enhanced mTOR signaling, further destabilizing the Treg phenotype. When overly active, mTORC1 can impair Treg suppressive function. mTOR signaling pathway and its relation to Treg function is further explained in the next section.

While CRL5-mediated degradation of pJAK1 is the primary mechanism of IL-2R desensitization, an alternative pathway of desensitization involves NOTCH signaling. In this mechanism, ASB2 (a SOCS family member) and SKP2 (an F-box protein) bridge CUL5 and CUL1 into the same complex that targets JAK3 for degradation (49). This suggests that IL-2 signaling in Tregs can be terminated not only through SOCS3–CRL5–mediated pJAK1 degradation, but also via NOTCH-driven ubiquitination of JAK3 through a CUL1/5 based complex.

Together, these pathways illustrate how neddylation of CRLs governs the intensity of IL-2R signaling in Tregs, and how dysregulation of these mechanisms may contribute to autoimmunity by disrupting pSTAT5 signaling and Treg suppressive function.

## 6 mTOR signaling pathways in Treg metabolic regulation

The mechanistic target of rapamycin (mTOR) is a serine/threonine kinase that regulates cell growth and metabolism. mTOR exists in two distinct complexes: mTOR complex 1 (mTORC1) and mTOR complex 2 (mTORC2).

While mTOR signaling is necessary for Treg development and function, excessive mTORC1 activation compromises Treg stability and suppressive capacity. mTORC1 promotes glycolysis and anabolic metabolism, supporting proliferation in effector T cells (Teffs) (43). In contrast, Tregs exhibit a more quiescent metabolic profile, relying on oxidative phosphorylation and lipid metabolism to maintain their suppressive phenotype (50). This distinction underscores the importance of limiting mTORC1 activity in Tregs to prevent phenotypic reprogramming toward a Teff-like state.

mTORC2 regulates Akt signaling and contributes to Treg survival and migration, though its role is context-dependent and less well defined (51). While moderate mTORC2 activity supports Treg function, hyperactivation may still impair their suppressive phenotype, acting as a metabolic rheostat that must be carefully balanced.

Tregs inherently express lower levels of mTOR activity compared to Teffs. This is maintained by FOXP3-dependent transcriptional programs, including upregulation of Pim-2, a growth kinase that functions independently of mTOR. Additionally, Tregs also upregulate PTEN which inhibits the PI3K/AKT pathway and further suppresses mTOR activity.

mTOR activity also feeds back to regulate post-translational pathways critical to Treg function. For example, mTOR activation promotes expression of Otubain-1 (Otub1), a deubiquitinase (DUB) that destabilizes GRAIL by deubiquitinating GRAIL (52, 53). Deubiquitination of GRAIL blocks the binding of USP8 to GRAIL. USP8 is the DUB responsible for removing ubiquitin from GRAIL (54). A catalytically inactive version of Otub1, Otub1-ARF1, expressed in Tregs can bind GRAIL and block Otub1 binding, thus allowing USP8 to function as a deubiquitinase to preserve GRAIL expression (52). This Otub1–USP8–GRAIL axis provides a mechanism by which increased mTOR activity further impairs IL-2 signaling by enhancing GRAIL degradation.

GRAIL also inhibits CRL1-mediated degradation of DEPTOR, an mTORC1 inhibitor, thereby enforcing Treg metabolic quiescence. In GRAIL-deficient states, DEPTOR is degraded, leading to mTORC1 activation, metabolic reprogramming towards a glycolysis-dependent phenotype, and loss of suppressive function. This initiates a positive feedback loop, where mTOR activation upregulates Otub1, further degrading GRAIL and amplifying Treg instability (53).

## 7 Targeting the neddylation pathway for autoimmune disease

Autoimmune diseases result from a breakdown in self-tolerance, allowing the immune system to attack tissues in the body. Tregs play a central role in preventing this, thus a defect in Treg function can lead to the development of autoimmunity. Proper Treg function requires prolonged IL-2 signaling for the transcription of genes that activate the suppressive function of Tregs. In the case of deficient GRAIL expression, CUL5 neddylation results in premature IL-2R desensitization. Inadequate GRAIL levels lead to the ubiquitination and proteasomal degradation of IL-2R second messengers (pJAK1, pSTAT5) and the termination of IL-2R signaling.

We hypothesize that loss of GRAIL and DEPTOR, coupled with increased Otub1 expression (that allow GRAIL degradation) in Tregs, may contribute to the pathology of multiple autoimmune diseases. In particular, we propose that reduced GRAIL expression leads to enhanced degradation of pJAK1 and DEPTOR, thereby disrupting IL-2R and mTOR signaling in Tregs. Premature IL-2R desensitization and excessive mTOR activation contribute to an unstable Treg phenotype which can play a role in the development of autoimmune disease. While low dose IL-2 therapy, to expand Tregs, has emerged as a potential treatment for multiple autoimmune diseases, a significant long-lasting therapeutic effect has not been observed, as this treatment does not correct the underlying signaling defect in Tregs (7, 18).

Due to the demonstrable possibility of delivering small molecules to the IL−2R via protein drug conjugates (55, 56), we developed both an IL−2 fusion protein and, more recently, an anti−CD25 antibody drug conjugate to deliver the NAEi to Tregs via their constitutively expressed high−affinity receptor for IL−2.

**Figure 4:**
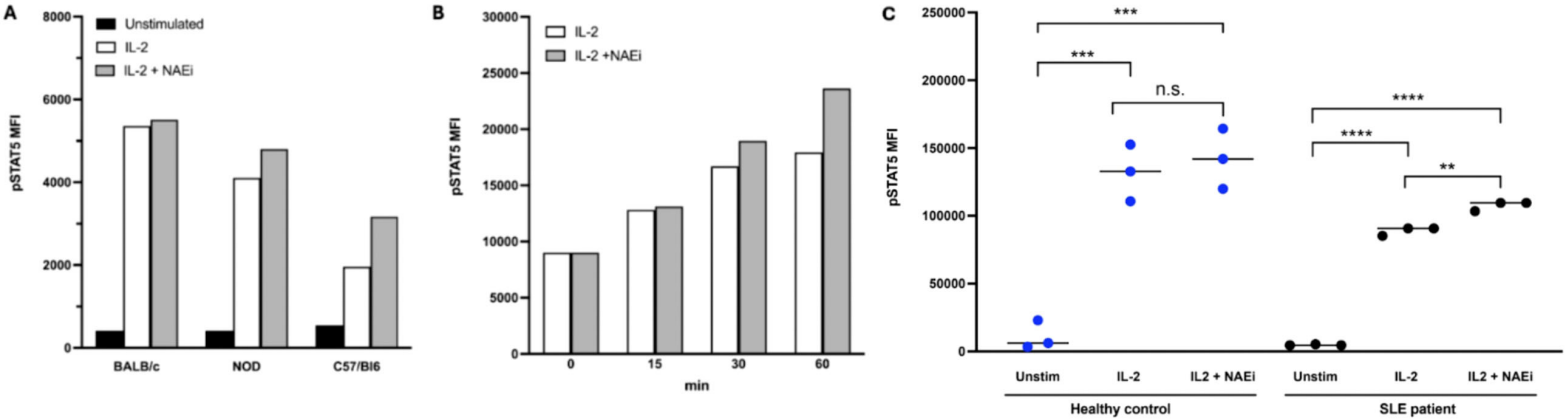
pSTAT5 expression in mouse and human Tregs was measured by FACS analysis. Combination treatment with low dose IL-2 (1 ng/ml) and NAEi (MLN4924, 1uM) enhanced IL-2 stimulated pSTAT5 expression in CD4+CD25+ Tregs of NOD (model of T1D) and C57/BL6 (auto-immune-prone) mice after 30 mins of treatment **(A)**. Combination treatment for 15–60 mins also enhanced pSTAT5 expression in Tregs of NZBWF1 [model of SLE; **(B)**] compared to treatment with IL-2 alone. In Tregs from healthy human controls and SLE patients studies *in in vitro* regulation analysis, treatment with a combination of low-dose IL-2 (1ng/ml) and NAEi (MLN4924; 1 uM) for 60 mins increased pSTAT5 expression compared to treatment with low dose-IL-2 treatment alone **(C)** (**P<0.005, ***P<0.001, ****P<0.0001, Student’s unpaired t-test).

Our lab has explored the use of neddylation inhibitors (NAEi) such as MLN4924 (aka TK924), in combination with low-dose IL-2, as a potential therapy for correcting the functional Treg defect. NAEi mimic GRAIL function, by covalently binding NEDD8, thus preventing the neddylation of CUL5 and likely CUL1 (57). This allows for the inhibition of IL-2R desensitization, even when GRAIL levels are low. As such, we have shown that IL-2 stimulated pSTAT5 activity is enhanced in both mouse and human Tregs when treated with NAEi (**Figure 4**), and that combination therapy with IL-2 and NAEi can reduce disease severity and arrest disease progression in animal models of autoimmunity (58).

While NAEi were found to have a therapeutic effect in clinical trials for cancer therapy, systemic treatment required high doses that were associated with significant toxicity due to off-target effects. Even at low concentrations, NAEi can impact CRL activity in various types of cells (36). This prompted us to develop protein drug conjugates (PDCs), and more recently antibody drug conjugates (ADC) that target the IL-2R to avoid non-specific toxic side-effects. Using the PDC strategy, the neddylation inhibitor is linked to an IL-2 fusion protein via a cleavable linker, enabling selective delivery of the NAEi to IL-2R–expressing Tregs. Upon receptor engagement and internalization, the drug is released through receptor-mediated endocytosis and cathepsin cleavage of the NAEi to exert its effect (18). In the ADC approach, the NAEi is conjugated to an anti-CD25 antibody that targets the high affinity IL-2R on Tregs. After binding and endocytosis, the drug is released in the lysosome, where it becomes active within the Treg. Both methods target the NAEi to Tregs by binding to the constitutively expressed high affinity IL-2R. This allows significantly lower amounts of drug to be used and erases the systemic toxicity of off-target effects.

**Figure 5:**
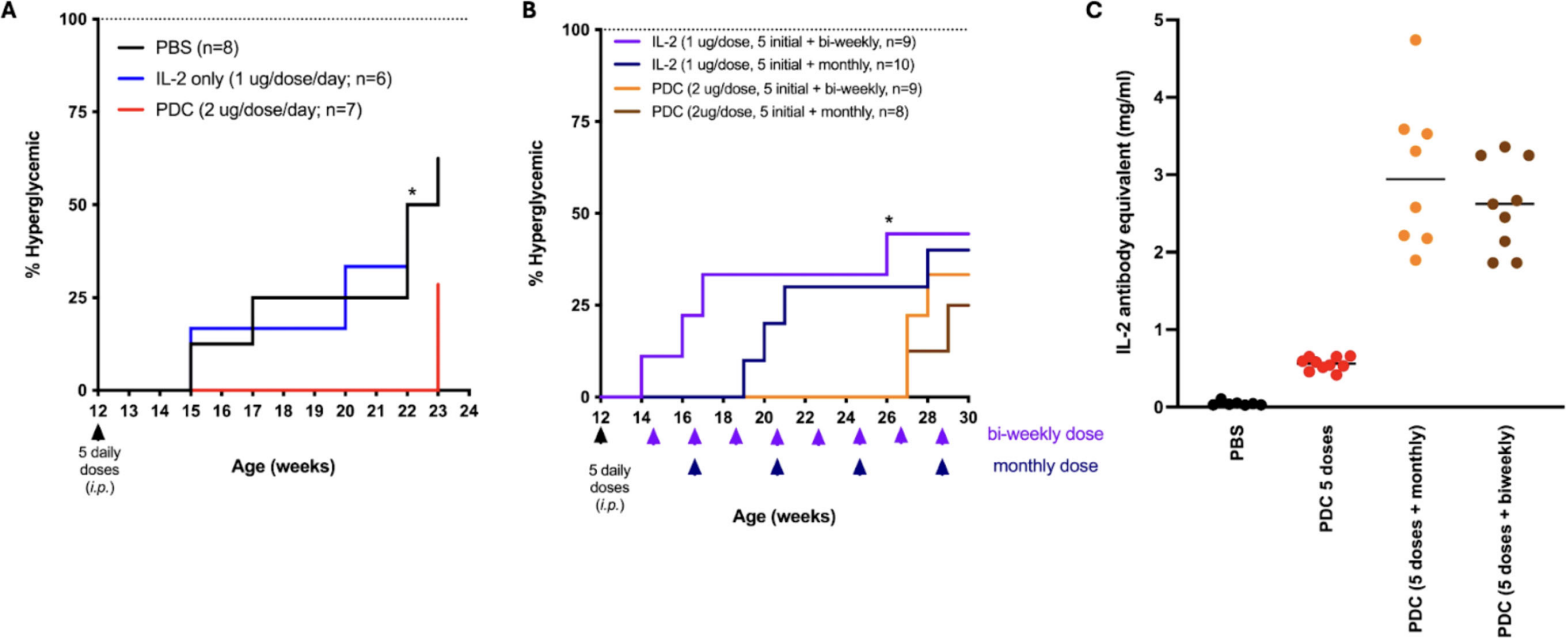
Non-obese diabetic mice treated with 5 daily doses of PDC had a significant 8 week delay in the onset of hyperglycemia compared to mice treated similarly with PBS or IL-2 **(A)**. Treatment with additional maintenance doses of PDC once every 2 weeks (bi-weekly) or every month delayed the onset of hyperglycemia for another 4 weeks **(B)**. The PDC was not able to fully prevent disease onset, likely due to the development of anti-drug antibodies that were detected in the serum of PDC-treated mice **(C)**.

The mouse PDC, developed, in collaboration with IL-2Rx, consists of a mouse thioredoxin-mouse IL-2 fusion protein conjugated to 3 molecules of MLN4924. This PDC was shown to be both safe and efficacious in delaying disease progression or reducing disease severity across multiple animal models of autoimmune and inflammatory diseases including SLE, T1D, MS, and asthma (18). Treatment with the PDC was effective in delaying the onset of hyperglycemia in the NOD mouse model of T1D (**Figure 5A**). However, maintenance therapy of the PDC, administered every 2 weeks or every month, was unable to prevent disease development (**Figure 5B**). Testing of serum collected from the PDC-treated mice showed that this was likely due to the development of anti-drug antibodies, likely against a novel epitope in the fusion protein (**Figure 5C**) as the PDC developed in our lab formed aggregates that may have been antigenic. These results led us to begin developing an ADC to allow targeting of the NAEi to Tregs using anti-CD25 antibodies. This strategy aims to enhance efficacy while minimizing systemic toxicity associated with untargeted NAEi delivery.

**Figure 6:**
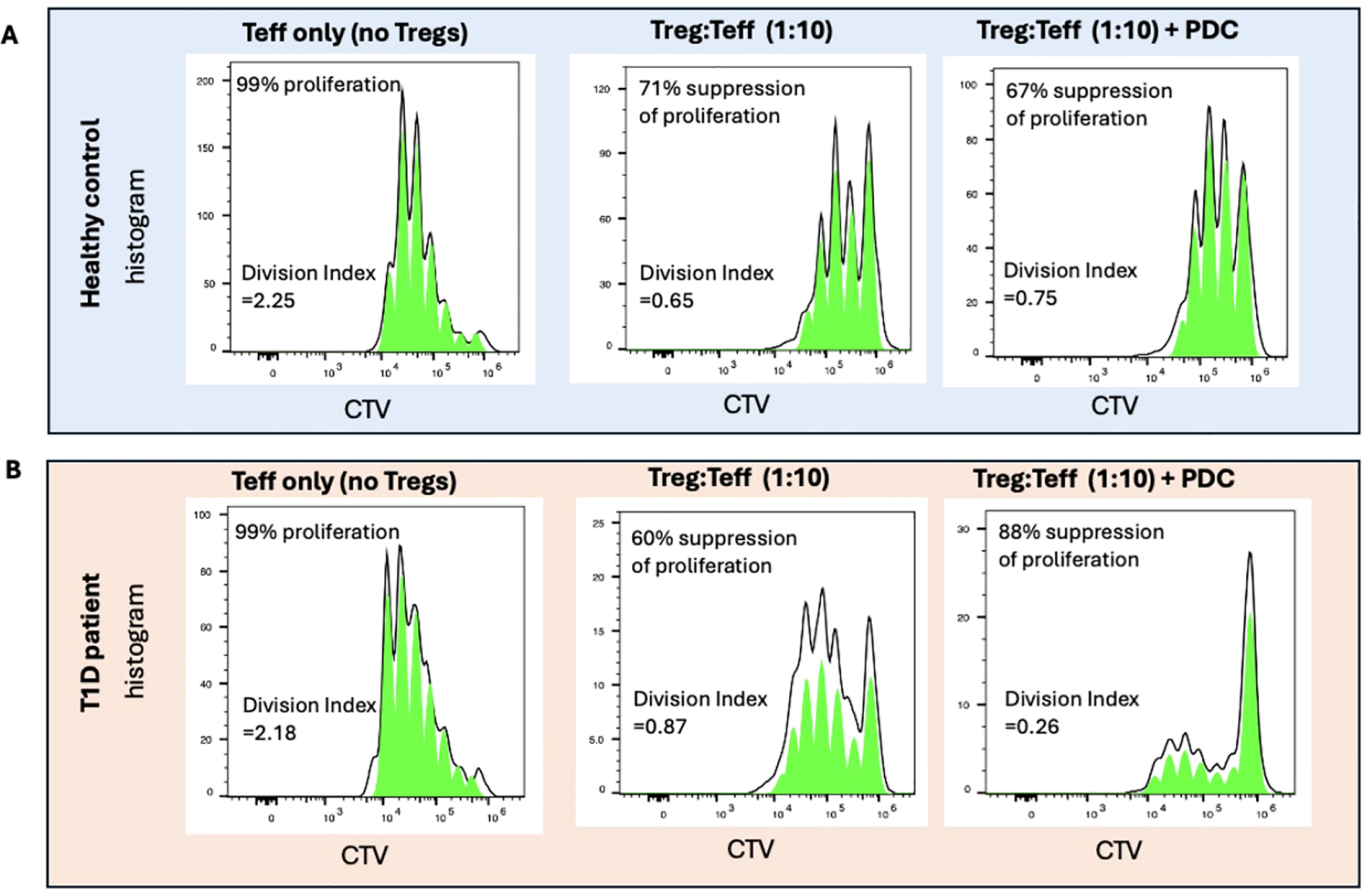
Treg suppression assays were performed using Teff cells labelled with the proliferation dye Cell Tracer Violet (CTV) and activated with anti-CD3/28 Dynabeads at ratio of 1:32 (beads:Teffs) for 5 days. As Teff cells proliferate, the CTV intensity decreases. In the absence of Tregs, 99% of Teffs from both healthy control and T1D patients were found to proliferate (left panel). When co-cultured with Tregs expanded *in vitro* (at a ratio of 1 Treg:10 Teffs), 71% suppression of proliferation was found to occur in a representative healthy control. Addition of PDC did not significantly change the suppressive effect of Tregs from healthy individuals **(A)**. The expanded Tregs of a representative T1D patient showed 60% suppressive activity when co-cultured with Teffs (1:10). In the presence of PDC, the suppressive capability of T1D Tregs were boosted to levels even higher than the healthy controls **(B)**. Percent of suppression was calculated by 100-[DI(Treg+Teff)/DI (Teff only)]x100. DI (Division Index) was generated by Flowjo.

While not ideal for studies *in vivo*, the PDCs were still useful for studies of Treg function *in vitro*. Using a human PDC consisting of a human thioredoxin-human IL-2 fusion protein conjugated to 3 molecules of MLN4924, we were able to show that treatment with PDC could restore the suppressive function of T1D patient Tregs that normally exhibit significantly reduced suppressive function compared to Tregs of healthy controls (**Figure 6**).

**Figure 7:**
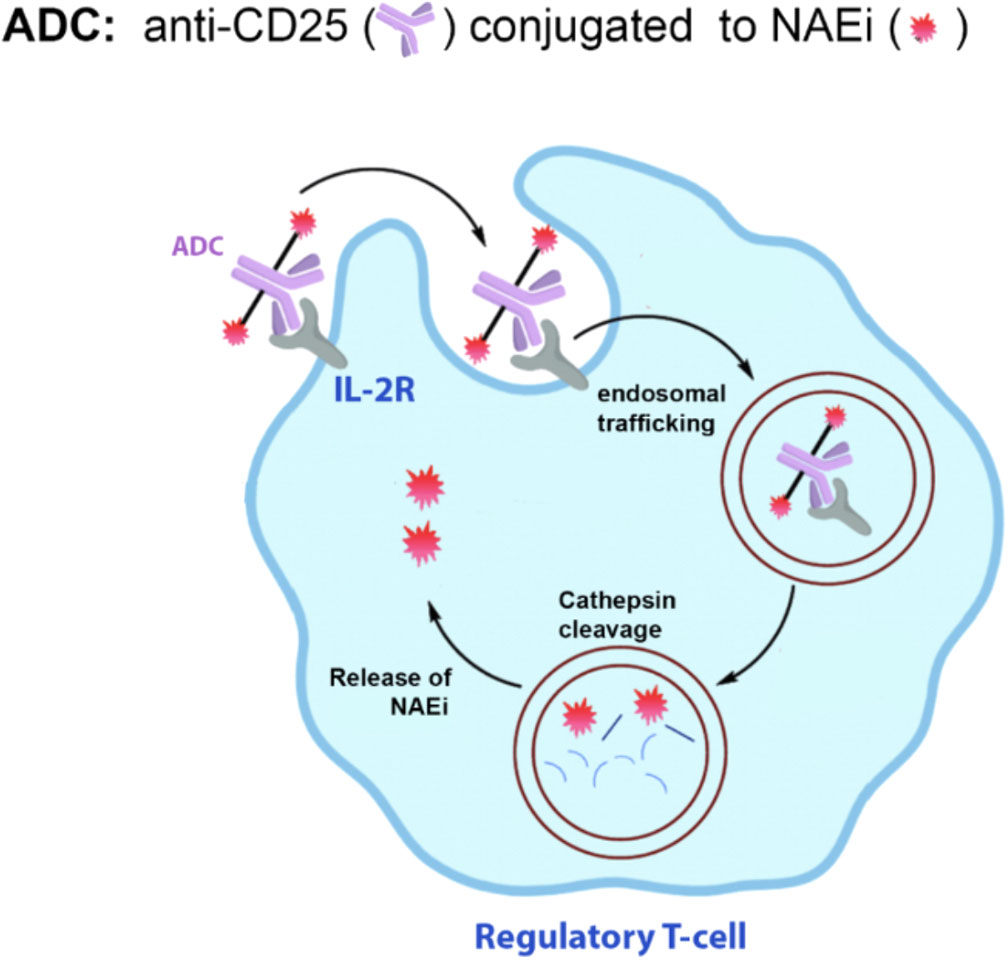
Proposed ADC to deliver NAEi to Tregs. NAEi will be conjugated to an anti-CD25 monoclonal antibody to allow targeting of the ADC to Tregs. Once the ADC binds to IL-2R, it will be internalized and shuttled to the endosome, where the drug is cleaved off the antibody by cathepsin B localized in the late endosomes.

Similar to PDCs, the ADCs should target Tregs to enhance efficacy and minimize systemic toxicity. The mouse ADC being developed in our lab consists of an anti-mouse CD25 monoclonal antibody conjugated to one or more molecules of MLN4924. CD25 is the third chain (high affinity chain) of the IL-2 receptor that is highly expressed on the surface of Tregs but not Teffs, making it an attractive target for selective delivery into Tregs without significantly affecting Teff cells (59). Once bound to CD25, the ADC will be internalized via receptor-mediated endocytosis, where it undergoes proteolytic cleavage in the lysosome to release the NAEi (**Figure 7**). Studies have shown internalization of a CD25-targeted ADC by time-dependent loss of surface-bound ADC and reduced membrane-associated fluorescence (60). This provides a strong proof-of-concept that ADCs can be used to target Tregs and regulate the immune microenvironment.

Our studies demonstrate that Treg activity is maintained by the balance of neddylation and ubiquitination at the Lys724 site of CUL5 in CRL5 and Lys720 in CUL1. Changes in GRAIL expression, such as diminished GRAIL expression seen in the Tregs of autoimmune patients, favor the neddylation of Lys724 of CUL5 and Lys720 of CUL1, and result in the degradation of IL-2R second messenger proteins and diminished Treg function. We have shown that NAEi can compensate for the loss of GRAIL by blocking neddylation at Lys724. Furthermore, we have shown that NAEi can be made into PDCs or ADCs by conjugating the NAEi to IL-2 fusion proteins or anti-CD25 monoclonal antibodies, respectively. This allows direct delivery of the NAEi to Tregs. We believe that NAEi, used alone or in combination with IL-2, as PDCs or ADCs, could serve as potential “off the shelf” drugs for correcting the signaling defect seen in the Tregs of autoimmune patients, and should induce a longer lasting therapeutic effect than that elicited by the expansion or adoptive transfer of Tregs.

## 8 Discussion

Treg dysfunction in autoimmune disease is increasingly recognized as a consequence of signaling defects rather than simple loss of Treg numbers. One such defect involves the dysregulation of post-translational modification of CRL5, which alters the balance between neddylation and ubiquitination within the IL-2R signaling pathway.

In Tregs from autoimmune patients, reduced expression of GRAIL allows neddylation of CUL5 to proceed, activating CRL5 and promoting degradation of pJAK1. This results in premature desensitization of the IL-2R, attenuated pSTAT5 activity, and impaired transcription of genes required for Treg suppressive function.

GRAIL and CRL5 act as antagonistic forces at the same lysine residue on CUL5 (Lys724). GRAIL mono-ubiquitinates this site, preventing neddylation and thereby inhibiting CRL5-mediated termination of IL-2R signaling. This competition forms a regulatory checkpoint that is critical for maintaining immune tolerance. Diminished GRAIL function disrupts this checkpoint, contributing to Treg instability and autoimmunity.

Strategies that mimic GRAIL’s function of inhibiting neddylation have shown promise in restoring Treg suppressive activity. Neddylation-activating enzyme inhibitors (NAEi) prevent NEDD8 conjugation to both CUL5 and CUL1, maintaining pSTAT5 signaling and blocking DEPTOR degradation thus preserving Treg function, even in the context of GRAIL deficiency. However, systemic administration of NAEi has been limited by toxic off-target effects, necessitating more selective delivery strategies. Targeted approaches using protein or antibody drug conjugates offer a promising solution by directing NAEi specifically to Tregs via the high-affinity IL-2R (CD25) expressed constitutively by Tregs. This allows for lower dosing, reduced off target exposure, and selective restoration of IL-2R signaling in dysfunctional Tregs.

Collectively, these findings support a model in which Treg activity is governed by the dynamic interplay between neddylation and ubiquitination at a single molecular site and suggests that modulating this axis may restore immune tolerance without broad immunosuppression. Restoring post-translational regulation of IL-2R signaling could represent a transformative strategy for treating a wide range of autoimmune diseases by targeting core signaling defects controlling Treg function.

## Data Availability

The original contributions presented in the study are included in the article/supplementary material. Further inquiries can be directed to the corresponding author.
